# Identification of binding sites and favorable ligand binding moieties by virtual screening and self-organizing map analysis

**DOI:** 10.1186/s12859-015-0518-z

**Published:** 2015-03-21

**Authors:** Emna Harigua-Souiai, Isidro Cortes-Ciriano, Nathan Desdouits, Thérèse E Malliavin, Ikram Guizani, Michael Nilges, Arnaud Blondel, Guillaume Bouvier

**Affiliations:** 1 Institut Pasteur, Unité de Bioinformatique Structurale, CNRS UMR 3528, Département de Biologie Structurale et Chimie, 25, rue du Dr Roux, Paris, 75015 France; 2 Laboratory of Molecular Epidemiology and Experimental Pathology – LR11IPT04, Institut Pasteur de Tunis, Université Tunis el Manar – Tunisia, 13, Place Pasteur, Tunis, 1002 Tunisia; 3 University of Carthage, Faculty of sciences of Bizerte – Tunisia, Jarzouna, 7021 Tunisia

**Keywords:** Self-organizing maps, Binding site, Chemical fingerprints, Chemical fragments, Virtual screening, Probe-mapping, Docking

## Abstract

**Background:**

Identifying druggable cavities on a protein surface is a crucial step in structure based drug design. The cavities have to present suitable size and shape, as well as appropriate chemical complementarity with ligands.

**Results:**

We present a novel cavity prediction method that analyzes results of virtual screening of specific ligands or fragment libraries by means of Self-Organizing Maps. We demonstrate the method with two thoroughly studied proteins where it successfully identified their active sites (AS) and relevant secondary binding sites (BS). Moreover, known active ligands mapped the AS better than inactive ones. Interestingly, docking a naive fragment library brought even more insight. We then systematically applied the method to the 102 targets from the DUD-E database, where it showed a 90% identification rate of the AS among the first three consensual clusters of the SOM, and in 82% of the cases as the first one. Further analysis by chemical decomposition of the fragments improved BS prediction. Chemical substructures that are representative of the active ligands preferentially mapped in the AS.

**Conclusion:**

The new approach provides valuable information both on relevant BSs and on chemical features promoting bioactivity.

**Electronic supplementary material:**

The online version of this article (doi:10.1186/s12859-015-0518-z) contains supplementary material, which is available to authorized users.

## Background

Identifying druggable cavities or pockets on a target protein is of high importance in the development of novel strategies in a structure-based drug discovery process. Binding sites (BSs), with or without ligand, are usually referred to as cavities at the protein surface and display a large variety of size and shape [1,2]. Consequently, in the context of drug discovery, refined criteria are necessary to discriminate potent binding pockets. The required properties, together referred to as “druggability”, are the subject of active research, and many scores have been elaborated to estimate them [3-5]. Protein-ligand interactions that promote binding appear to be mainly driven by cavity shape and size, as well as by chemical complementarity between the ligand and the protein atoms.

Existing methods and algorithms typically use evolutionary, geometrical, probe-mapping or energy-based principles for BS identification. Evolutionary methods [6-8] make use of structure and/or sequence alignments to identify BSs. They assume that conserved residues among one group of functionally related proteins would vary across different groups [9] so they constitute an “evolutionary trace” of BSs. These approaches are limited by the fact that conserved features may not be correlated to protein activity but rather to stability or folding [10]. Moreover, as a consequence of a low degree of sequence similarity or identity within a working dataset for a given protein query, the obtained results may be poor [10]. Purely geometric methods [1,11-15] have the advantage of being fast. They assume that a BS is a cavity or a cleft in the receptor surface and do not model the potency of a detected cavity to bind a drug-like molecule. Consequently, they can not distinguish different types of sites (e.g., hydrophobic versus polar). Finally, probe-mapping [16-18] and energy-based [2,19] methods are most of the time coupled (e.g., SILCS [20]). They calculate the energy between a probe and the target on a grid and in this way map energetically favorable areas for binding. Probes can be atoms (aliphatic carbon, aromatic carbon, hydrogen, oxygen, nitrogen, sulfur, etc) [2,18] or functional groups (methyl, amine, hydroxyl, cetone groups, etc) [19,21,22]. Many studies make simultaneous use of two types of approaches (geometric, energy-based, probe-mapping or evolutionary methods) or are coupled with other computational strategies. For instance, combining geometrical and energy based principles, through the “MetaPocket” server [23], improved the accuracy of these methods. Bowman [24] and Meagher [25] used receptor flexibility to successfully identify pharmacophores, used as probes, that are highly present in known inhibitors of the targeted protein. In a recent work, Glinca and Klebe [26] showed that the use of exposed physicochemical properties on cavities is more valuable than the use of sequence information, in the classification of protein families with respect to inhibitor selectivity. This stresses importance of considering protein-ligand interactions on the energetic level to assess a pocket’s “druggability”.

Probe-mapping and energy-based methods are the obvious way to model chemical complementarity between the ligand and the protein atoms. PocketFinder [2], for example, assesses a van der Waals potential over a grid and identifies all pockets with a volume larger than 100Å ^3^. In 80.9% of the cases, 50% of the ligand overlapped the largest pocket and 11.8% overlapped the second one. Q-siteFinder [19] uses GRID [16] to calculate a van der Waals potential of a methyl probe. Probes with favorable interaction energies are clustered. Clusters are then ranked according to their total interaction energies and the top 3 are considered as binding pockets. In 90% of the cases, 25% of the active ligand atoms were within 1.6Å of one of the top ranked pockets. An algorithm similar to Q-SiteFinder, called SiteHound [27], uses AutoGrid from the AutoDock 4 suite [28] for grid calculation and pocket prediction instead of GRID [16]. Then, after docking a known ligand on 77 proteins, the authors found that in 95% of the cases, the ligand center falls within 10.0Å of at least one of the first three predicted sites. The SiteHound success rate varied between 80 and 84% when the criterion was set to 15% or more ligand heavy atoms within a radius of 2.0Å from one of the first three predicted sites. Another algorithm called FTSite [22] performs a global search of the protein surface for regions that bind small organic probes by making use of a fast Fourrier transform approach [21]. FTSite was tested on the test set used by the Q-siteFinder authors [19] and performed at a success rate of 97% with same parameter values (precision = 25%, radius = 1.6Å) [22].

The present work introduces a new concept for the identification of BSs. It directly uses docking calculations, in combination with an analysis of the results by Self-Organizing Maps (SOMs) [29]. The SOM algorithm has many applications and can virtually be applied on any type of data. For example, SOMBRERO [30,31] is a SOM-based algorithm that detects transcription factor BSs on DNA sequences. A spherical SOM (SSOM) appeared useful in mapping a protein surface onto a sphere to better characterize its active site [32]. SOMs have a wide range of uses in virtual screening analysis and hit selection [33-35]. A recent paper used SOM as a tool for identifying macromolecular targets of de-novo designed chemical entities [36]. Digles [37] presents an interesting review of this specific application of SOMs. We have recently demonstrated the usefulness of SOMs in the analysis of molecular dynamics trajectories and ligand docking poses [38-41].

In a first step, we calibrated our method on two challenging targets from the “Database of Useful Decoys - Enhanced” (DUD-E) [42]. The DUD-E database provides dedicated ligand libraries for each protein target to benchmark docking approaches, one with the known effectors, and one with a series of decoy compounds. We also used an additional “generic” library, the Enamine Golden Fragments (EGF) collection (www.enamine.net). It contains a moderate number of entities, 1500 fragments, presenting a wide chemical diversity (see Additional file 1: Figure S1). The use of this library permits us to cover a large chemical space with a limited computational effort. Blind dockings of these databases were performed with two free software packages that have been extensively used and evaluated by others [43-47]; Dock [48] and AutoDock Vina (ADvina) [49], and are based on different searching algorithms and scoring functions. We identified the combination of docking program and ligand library giving the best prediction rates in this analysis. Then, we used this combination to assess the method accuracy in BS identification on all targets in the DUD-E (102 proteins). Docking results were analyzed with an in-house version of the Self-Organizing Map (SOM) applied directly to the ligand atomic coordinates as descriptors. The resulting SOMs provide a simple and intuitive representation of the spatial distribution of the docking poses. BSs can be identified as zones of high docking pose density and homogeneity.

In addition, we tested whether the proposed approach could give some “a priori” information on the chemical nature of potential ligands. For that, we analyzed the chemical structure of the docked compounds from the naive fragment library with Morgan fingerprints, which provide a decomposition of the molecules into a set of “chemical features” [50,51]. Previous studies have shown the efficiency of circular fingerprints in drug discovery tasks, such as the search for ligand analogues or virtual screening [52-54]. With SOM, we analyzed how the geometrical centers of these chemical features are distributed in space upon docking. Interestingly, this analysis provided an even more accurate mapping of the BSs, thus enhancing the interpretability of the SOMs. Furthermore, the chemical features of the naive library that are also present in active ligands mapped preferentially in the active sites.

## Methods

### Protein targets and ligand libraries

The DUD-E database [42,55] provides 102 targets ready for docking in pdb format. For each target, a definition of the active site (AS) is provided by means of the co-crystal 3D structure of the target with an active ligand. Prior to the assessment of the method’s accuracy in BS identification, we tuned the parameters of the approach to obtain the most accurate predictions on two specific targets.

**Figure 1:**
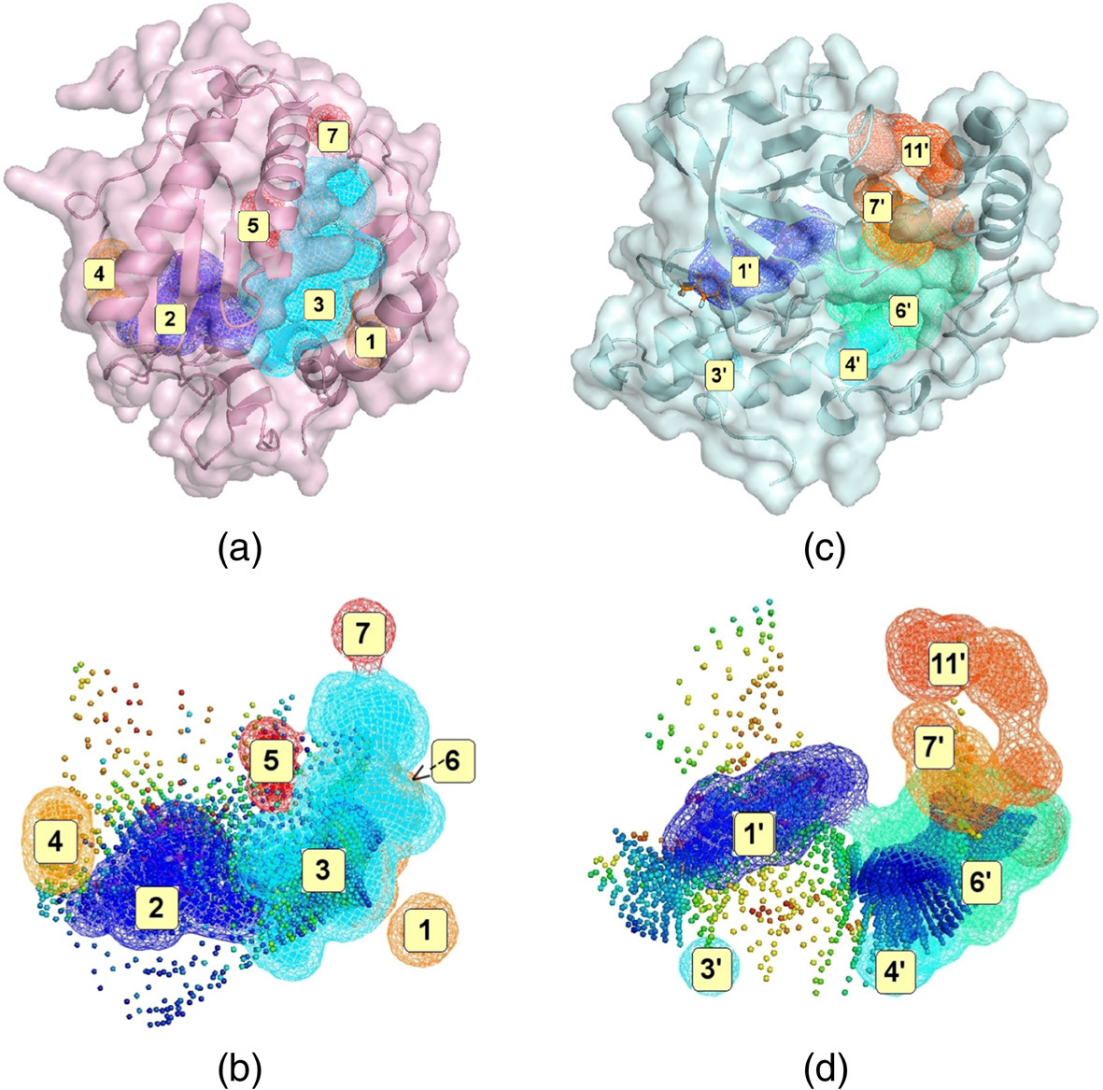
**Cavities detected with** ***mkgrid*** **and mapping of the docking outputs with SOMs for DUD-E active molecules docked with ADvina.** **(a)** HIV-RT represented by ribbons and pink transparent surface, with cavities labeled (1,2,3,4,5,7). Cavities 6,8 and 9 are not visible on this 2D projection. **(b)** SOM representation of results. AS fits in the dark blue cavity (2) and BS2 in the big cyan cavity (3). Cavity (6), behind, is pointed out with an arrow. **(c)** ABL1 represented by ribbons and light blue transparent surface, with cavities labeled (1’,3’,4’,6’,7’ and 11’). The remaining cavities are not visible on this 2D projection. **(d)** SOM representation of results. AS fits in the dark blue cavity (1’) and BS2 is in cavity (6’).

The first target, the HIV-1 reverse-transcriptase, is a heterodimer with two structurally distinct subunits, p51 (429 AA) and p66 (553 AA) [56]. The docking target site defined in the DUD-E is a sub-domain (272 AA) derived from the 3LAN PDB entry [57]. It is composed of a part of p66 and a small portion of p51 and contains the active site, an allosteric site and many other pockets and cavities (Figure 1). The DUD-E provides 338 active molecules and 18880 decoys for HIV-RT.

The second target is a sub-domain (221 AA) of the human tyrosine-protein kinase ABL1 (1130 AA) as defined in the DUD-E (PDB entry: 2HZI) [58]. Similarly to the first target, this sub-domain contains the active site, a secondary BS and many other cavities (Figure 1). A library of 182 actives molecules and 10745 decoys is provided for ABL1.

In addition to the specific libraries of active and non-active molecules provided by the DUD-E, we docked the Enamine Golden Fragment (EGF) library, composed of 1500 fragments, (www.enamine.net). The EGF collection follows the “Rule of Three” [59] with range of values in slightly tighter intervals. In practice, these ranges are: (i) molecular weight within [150,300] Da; (ii) clogP within [−2,3]; (iii) less than 3 Hbond acceptors; (iv) less than 3 Hbond donors; (v) less than 3 rotatable bonds and (vi) polar surface area inferior to 60Å^2^.

### Cavity identification

An in-house software based on the Lee and Richards solvent accessible surface calculation algorithm [60], called *mkgrid* [61] was used to detect cavities embedded in both protein targets. The method discretizes space on a 0.5 Å grid and calculates the solvent accessible volume with a 1.4 Å radius probe sphere (also accessing interior cavities). Bulk solvent is defined with a 10 Å radius probe sphere. Cavities are defined as the volume accessible to the solvent, but not to bulk solvent. Remaining void grid points are clustered by connectivity and labeled according to their cluster number to identify individual cavities. Clusters having less than 96 points (12 Å ^3^, about the volume of a water molecule) are discarded. The cavities were graphically inspected.

### Docking & virtual screening

The Dock6.0 (Dock) [48] and AutoDock Vina (ADvina) [49] programs were used for docking. The clustering step during pruning of the anchor and grow incremental construction approach was disabled for dock. Otherwise the default parameters were used. For Dock, receptor files were prepared with Chimera (www.cgl.ucsf.edu/chimera) [62]. Hydrogens were removed, Gasteiger charges calculated and molecular surfaces generated. We used the spheres, docking box and mol2 ligand files provided by the DUD-E. For ADvina, the required PDBQT files for the receptor and the ligands were generated from the original mol2 files with the Open Babel converter (openbabel.org). A maximum of 20 lowest-energy poses were kept for each ligand.

### SOM

**Figure 2:**
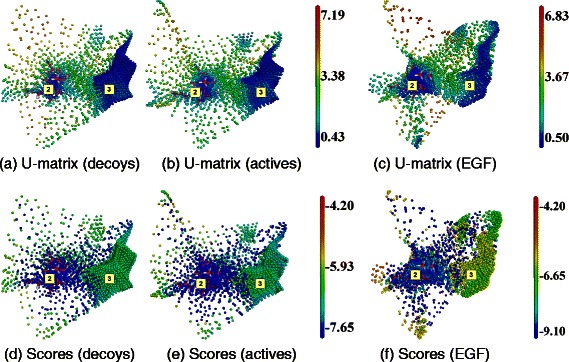
**SOM analysis of docking results obtained with ADvina on HIV-RT.** Top line, **(a)**, **(b)** and **(c)**: U-matrices; bottom line, **(d)**, **(e)** and **(f)**: docking score projections. Left column, **(a)** and **(d)**: DUD-E decoys set (map dimensions (I,J,K) = (24,13,11)). Middle column, **(b)** and **(e)**: DUD-E active molecules ((I,J,K) = (21,14,11)). Right column, **(c)** and **(f)**: EGF collection ((I,J,K) = (21,15,11)). Labels (2) and (3) correspond to cavity numbers used in Figure 1. They designate the AS and BS2, respectively.

**Figure 3:**
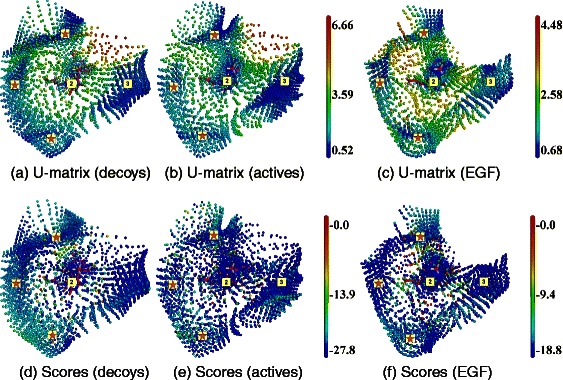
**SOM analysis of docking results obtained with Dock on HIV-RT.** Top line, **(a)**, **(b)** and **(c)**: U-matrices; bottom line, **(d)**, **(e)** and **(f)**: docking score projections. Left column, **(a)** and **(d)**: DUD-E decoys set (map dimensions (I,J,K) are (23,17,8)). Middle column, **(b)** and **(e)**: DUD-E active molecules ((I,J,K) = (23,18,8)). Right column, **(c)** and **(f)**] EGF collection ((I,J,K) = (21,17,9)). Labels (2) and (3) correspond to cavity numbers used in Figure 1. They designate the AS and BS2, respectively. Regions labeled with red stars correspond to SOM regions appearing on the HIV-RT surface and considered as docking artifacts.

**Figure 4:**
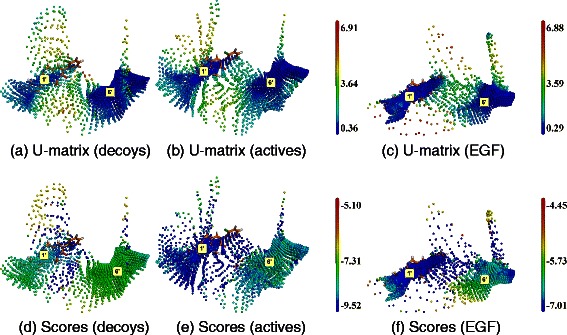
**SOM analysis of docking results obtained with ADvina on ABL1.** Top line, **(a)**, **(b)** and **(c)**: U-matrices; bottom line, **(d)**, **(e)** and **(f)**: docking score projections. Left column, **(a)** and **(d)**: DUD-E decoys set (map dimensions (I,J,K) are equal to (28,15,8)). Middle column, **(b)** and **(e)**: DUD-E active molecules ((I,J,K) = (31,14,8)). Right column, **(c)** and **(f)**: EGF collection ((I,J,K) = (34,12,8)). Labels (1’) and (6’) correspond to cavity numbers used in Figure 1. They designate the AS and BS2, respectively.

**Figure 5:**
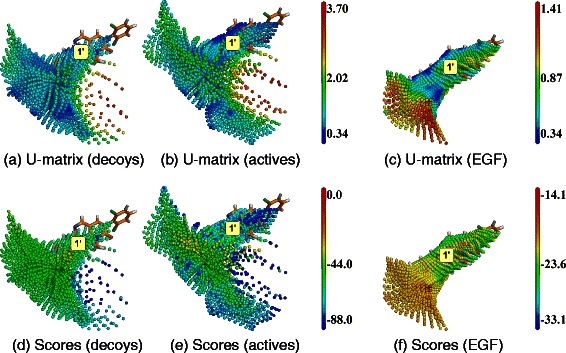
**SOM analysis of docking results obtained with Dock on ABL1.** Top line, **(a)**, **(b)** and **(c)**: U-matrices; bottom line, **(d)**, **(e)** and **(f)**: docking score projections. Left column, **(a)** and **(d)**: DUD-E decoys set (map dimensions (I,J,K) are (18,17,11)). Middle column, **(b)** and **(e)**: DUD-E active molecules ((I,J,K) = (19,18,10)). Right column, **(c)** and **(f)**: EGF collection ((I,J,K) = (28,13,9)). The label (1’) corresponds to the cavity number used in Figure 1. It designates the AS.

To analyze the docked ligand poses, we used an in-house implementation of the Self-Organizing Map (SOM) algorithm first introduced by Kohonen [29]. We trained a 3D non-periodic map, *Ω*_*ijk*_, with the *n* 3D coordinate of all atoms of all retained docked ligand poses. To set up the SOM, the whole set of *n* atomic coordinates was analyzed by Principal Component Analysis (PCA). This yielded a set of three normalized principal components, *V*_*i*=1,2,3_, with associated lengths *S*_*i*=1,2,3_, the square roots of the eigenvalues. The dimensions of the SOM, I, J and K, were set to integer values approximately proportional to *S*_1_,*S*_2_,*S*_3_, with a product I ×*J*×K close to 15^3^. These map dimensions are given in the legends of the Figures 2, 3, 4 and 5 displaying the SOM results. The maximum and minimum projection values over the *n* input vectors on *V*_*i*=1,2,3_ were calculated as *V* *i*+ and *V* *i*−. The SOM was initialized with triplets of real numbers regularly spaced along the three eigenvectors: $\Omega _{\textit {ijk}} =\left (V_{1}^-+i. \left (V_{1}^+-V_{1}^-\right)/I\right)$. $V_1+\left (V_{2}^-+j.\left (V_{2}^+-V_{2}^-\right)/J\right).V_2+\left (V_{2}^-+k.\left (V_{3}^+- V_{3}^-\right)/K\right).V_{3}$.

A training cycle consisted in the presentation of each of the *n* input vectors in random order with an update of the SOM after each presentation (step). Two phases, *ϕ*=1,2, similar to those previously used [38] were carried out. In each phase, the radius *r*_*ϕ*,*t*_ and the learning rate *α*_*ϕ*,*t*_ at step *t* decreased exponentially between initial (0) and final (*f*) values, *r*_*ϕ*,0_ and *r*_*ϕ*,*f*_ respectively (*r*_*ϕ*,*t*_=(*r*_*ϕ*,0_−*r*_*ϕ*,*f*_)· exp(−*t*/*λ*_*ϕ*_)+*r*_*ϕ*,*f*_). The exponential decay, *λ*_*ϕ*_, was set to the total number of steps of the phase divided by 10. In the first phase, one training cycle of *n* steps was performed with ((*r*_1,0_,*r*_1,*f*_),(*α*_1,0_,*α*_1,*f*_))=((7.5,3.75),(1,0.5)). In the second phase, ten training cycles were performed with the parameters set to ((3.75,1),(0.5,0.1)).

As the SOMs were set up with 3D Cartesian coordinates, their spatial representation on the protein structures was straightforward. The average docking score of the atoms mapping one neuron, for example, could simply be displayed with a color code at the position specified by the neuron value.

An element of the Unified Distance Matrix, or U-matrix, formed by I ×*J*×K elements *U*_*i*,*j*,*k*_ called “U-values”, is calculated from the SOMs as the mean Euclidean distance of the neuron to its 26 direct neighbors. We call areas with low U-values: “high neuron consensus” areas.

### SOM analysis and BS identification

The SOM algorithm reveals docking hotspots by the presence of areas with low U-values and generally high neuron densities. Areas between docking hotspots appear as low density regions on the SOM, and associated with high U-values.

We defined a cutoff (*t*_*U*_) on the U-values to distinguish between potential BSs (consensual binding regions with U-values ≤*t*_*U*_) from barriers between BSs (regions with U-values >*t*_*U*_). To automate the definition of *t*_*U*_, we fitted a Gaussian mixture model (GMM) to the distribution of the U-values with an algorithm implemented in the scikit-learn python package [63]. The number of Gaussians to fit was defined by making use of the Bayesian information criterion (BIC) [64]. The components which had the largest Gaussian weight were selected. The threshold on the U-value (*t*_*U*_) was then defined as:

(1) $$ t_{U} = \mu_{U} + \sqrt{\sigma_{U}^{2}}  $$

where *μ*_*U*_ and $\sigma _{U}^{2}$ are the mean and the variance of the dominant Gaussian. Neurons with U-values ≤*t*_*U*_ were aggregated by connexity and defined *n*_*cc*_ consensual clusters (CCs).

A radius was then defined to assess if a ligand atom is overlapping a given CC. This radius was automatically set with the same strategy as used for *t*_*U*_. The distribution of distances to the nearest neighbors within the CC (4 neighbors per neurons except at the borders of the SOM) is fitted with a 2 components GMM. The cutoff distance, called radius, *r*_*CC*_, is then:

(2) $$ r_{CC} = \mu_{d} + \sqrt{\sigma_{d}^{2}}  $$

where *μ*_*d*_ and $\sigma _{d}^{2}$ are the mean and the variance of the dominant Gaussian. The precision is defined as the fraction of ligand atoms that are within *r*_*CC*_ distance from any of the CC neurons.

A SOM neuron was considered to be inside a given cavity, defined by *mkgrid* (see Cavity identification paragraph), if at least one corner of the grid cube encompassing its position value had the label of that cavity.

### Chemical descriptors

Compounds were decomposed into chemical substructures with the circular Morgan fingerprints algorithm [50,51] as implemented in RDkit [65], as these Fingerprints have proved efficient in virtual screening [52-54]. Fingerprints are calculated by decomposition of the compound into substructures with a user-defined maximal diameter (number of connected bonds). A unique integer identifier is then assigned to these substructures according to atom types and their neighbors.

Substructures were called “chemical features” here. We calculated features with diameters up to 7 non-hydrogen atoms and then filtered features with 3 to 7 non-hydrogen atoms. The docking analysis of the features was performed with SOMs by making use of the geometric center coordinates of each chemical feature as input vectors.

### Analysis of the results obtained with the “chemical features” decomposition

We defined the following sets: (i) *F*_*EGFd*_ is the set of chemical features present in the EGF compounds which were successfully docked at the protein surfaces, (ii) *F*_*AS*_ is the set of chemical features of the EGF compounds docked at the AS, (iii) $\overline {F_{\textit {AS}}}$ is the set of chemical features of the EGF compounds which could never dock at the AS, (iv) *F*_*A*_ the set of chemical features present in both the *F*_*EGFd*_ and the set of DUD-E active ligands of the considered target. This represented the set of “active features”. It was used as a validation set *a posteriori*.

Using |*F*| as the cardinal of the set *F*, i.e the number of features belonging to that set, we calculated the enrichment in active features for *F*_*EGFd*_, *F*_*AS*_ and $\overline {F_{\textit {AS}}}$ as follows:

(3) $$ E(EGFd) = \frac{| F_{A} | }{ | F_{EGFd} | }  $$

(4) $$ E(AS) = \frac{|F_{A} \cap F_{AS}| }{ |F_{AS}| }  $$

(5) $$ E(\overline{AS}) = \frac{|F_{A} \cap \overline{F_{AS}}| }{ |\overline{F_{AS}}|}  $$

Then, the sensitivity *Se* was calculated as the number of “active features” that docked in the AS divided by the total number of “active features”:

(6) $$ Se = \frac{|F_{A} \cap F_{AS}| }{ |F_A| }  $$

Similarly, the specificity *Sp* was calculated as the number of “inactive features” that never docked in the AS divided by the total number of “inactive features”:

(7) $$ Sp = \frac{| (F_{EGFd} \backslash F_A) \cap \overline{F_{AS}} | }{ | F_{EGFd} \backslash F_{A} | }  $$

where *F*_*EGFd*_∖*F*_*A*_ is the set of chemical features present in *F*_*EGFd*_ and not in *F*_*A*_, which constitutes the set of “inactive features”.

To assess the quality of these quantities, we built a null hypothesis by randomization of the features that dock in the AS (*F*_*AS*_) 1 million times. In a perfect scenario, all the active features (*F*_*A*_) would dock in the AS, giving a sensitivity equal to 1. In the worst scenario, none of the active features would dock in the AS and *Se* = 0. The results data should be normally distributed *N*(*μ*,*σ*). The Z-score is the distance in terms of *σ* between the sensitivity obtained and the mean *μ* of the normal distribution of sensitivities corresponding to a random distribution of the features. We performed the same analysis for the specificity by randomizing the features that would never dock in the AS ($\overline {F_{\textit {AS}}}$). We consider that any Z-score value higher than 4 for *Se* and *Sp* indicate strong significance as they could not have been obtained randomly.

## Results

### Binding site identification

We used *mkgrid* to calculate cavities for all 102 targets (see Additional file 1: Table S1), and classified them by the number of cavities having a volume superior to 100Å ^3^ (Table 1). We chose representative targets from the two largest categories of targets, which had two or three cavities larger than 100Å ^3^: ABL1 and HIV-RT, respectively. In a first step of the present work, we calibrated our method on these two targets.

**Table 1:** **DUD-E targets clustered into categories, according to the number of cavities (detected with** ***mkgrid*** **) with a volume superior to 100Å** ^**3**^

**Nbre cav**	**Targets**
8	pa2ga
6	hmdh
5	braf
4	reni prgr pgh2 glcm esr2 dpp4 cxcr4 cp3a4
3	mk01 kpcb kith **hivrt** esr1 drd3 cp2c9 aofb adrb1 aces pgh1 parp1
2	vgfr2 thrb thb tgfr1 src sahh pyrd pygm pparg ppard ppara nram mcr
	lck jak2 inha gria2 gcr fgfr1 dhi1 bace1 andr ampc adrb2 ace **abl1**
1	aa2ar ada17 ada akt1 akt2 aldr cah2 casp3 cdk2 comt csf1r def dyr
	egfr fa10 fa7 fabp4 fak1 fkb1a fnta fpps grik1 hdac2 hdac8 hivint hivpr
	hs90a hxk4 igf1r ital kif11 kit lkha4 mapk2 met mk10 mk14 mmp13 mp2k1
	nos1 pde5a plk1 pnph ptn1 pur2 rock1 rxra try1 tryb1 tysy urok wee1 xiap

The targets HIV-RT and ABL1 used for the calibration step are shown in bold. They belong to categories 3 and 2, respectively.

We analyzed the successful docking poses with SOMs applied on individual atom Cartesian coordinates. For HIV-RT, 337 out of 338 DUD-E active molecules could be docked with ADvina. Similarly, 18873 out of 18880 DUD-E decoys, and 1421 out of 1500 EGF fragments docked. With Dock, the numbers of successfully docked molecules were 194, 12273, and 1152, respectively. For ABL1, ADvina allowed the docking of all DUD-E active molecules (182), all decoys (10745) and 1421 out of 1500 EGF fragments. With Dock on ABL1, 180, 10674 and 1422 molecules docked, respectively. The maps are shown in Figures 2, 3, 4 and 5.

The distances of the input vectors to their representative neurons were calculated to evaluate the acuity of the SOMs (see Additional file 1: Figure S2). They were normally distributed. For HIV-RT, they were 0.33 ± 0.40 Å with ADvina and 0.50 ± 0.35 Å with Dock. For ABL1, distance distributions were similar for ADvina and Dock (0.33 ± 0.40 Å). Overall, the SOMs appeared fairly acute.

U-values and docking scores were projected on the SOMs and displayed with a color gradient. The co-crystallized ligand was also represented in licorice to show the AS position (Figures 2, 3, 4 and 5). Lower values (dark blue) indicate regions with a large number of docked molecules. These high neuron consensus regions are plausible BS candidates.

#### HIV-RT

The SOM analyzes for HIV-RT are shown in Figure 2 for ADVina, and Figure 3 for Dock. U-matrices are shown in the top line (a-c) and docking score projections in the bottom line (d-f).

For ADvina, the U-values for the three libraries, DUD-E decoys, DUD-E active molecules and EGF fragments, shown in Figure 2(a) to (c), are quite similar, with the same areas showing low U-values. One, labeled (2), contains the co-crystallized ligand, and thus fits the AS as described in the literature (PDB entry: 3LAN). The second area, labeled (3), corresponds to an allosteric site of HIV-RT [66] and will be referred to as the second binding site (BS2) of HIV-RT. For the EGF library, a higher neuron consensus (lower U-values) is observed at the AS than with the DUD-E active molecules. Inversely, the fragments gave lower neuron consensus than the DUD-E active compounds at the BS2. As regards to docking scores, they are more favorable at the AS than at the BS2 for all three libraries (Figure 2(a) to (c)).

The maps obtained with Dock have a different shape (Figure 3). Five clusters could be identified. Two of them correspond to the AS and the BS2 described above. The remaining clusters appear at the surface of the protein (marked by red stars on Figure 3), and did not match any detected cavities (Figure 1). They will not be considered as relevant BSs here.

The neuron consensus obtained at the AS with the DUD-E active molecules and the EGF fragments (Figure 3(b) and (c)), were higher than with the DUD-E decoys (Figure 3(a)). The docking scores alone could not provide any discrimination with neither of the three libraries (Figure 3(a) to (c)).

#### ABL1

The SOMs obtained on ABL1 with ADvina are shown in Figure 4. U-matrices revealed two high neuron consensus areas. The first one is the AS of ABL1, containing the co-crystallized ligand (PDB entry: 2HZI; Figure 4; label (1’)). The second area matches a big pocket labeled (6’). It is close to the AS and involves the activation loop of ABL1 [67] and will be referred to as the BS2 of ABL1.

Fragments from the EGF library yielded a more compact map than the DUD-E molecules (Figure 4(c)). The highest neuron consensus appeared at the AS.

The docking scores at the AS were lower than at the BS2 with the three libraries (Figure 4(a) to (c)). DUD-E active molecules and EGF fragments had better scores at the AS than the decoys.

The SOM analysis of Dock outputs are reported in Figure 5. Although the spheres defining the docking area cover the AS, BS2 and other pockets, molecules only docked in the AS.

DUD-E active molecules (Figure 5(b)) mapped the AS better than the decoys (Figure 5(a)), as denoted by lower U-values. The EGF fragments yielded an even more compact map (Figure 5(c)), tightly fitting the AS.

### Binding site characterization

For both targets, *mkgrid* detected cavities corresponding to AS and BS2, as well as other cavities (Figure 1). We detected 9 cavities in the HIV-RT target subdomain, 3 of which had a volume larger than 100 Å ^3^ (see Table 1 and Additional file 1: Table S2 for cavities labels). For the ABL1 subdomain these figures were 12 and 2, respectively. We calculated the neuron density as the number of neurons inside the cavity divided by the cavity volume (see Additional file 1: Table S2). We used the SOMs trained on the EGF outputs for that calculation.

#### HIV-RT

For HIV-RT, cavities number (2) and (3) corresponding to the AS and BS2, have volumes of 338.5 Å ^3^ and 957.4 Å ^3^, respectively. The EGF fragments yielded the highest neuron densities in the AS (3.070 and 2.065 neuron/Å ^3^ with ADvina and Dock, respectively; Table 2). DUD-E active molecules showed lower neuron densities (1.563 and 1.731 neuron/Å ^3^ with ADvina and Dock, respectively). The lowest values were obtained with the DUD-E decoys (1.158 and 0.718 neuron/Å ^3^ with ADvina and Dock, respectively).

**Table 2:** **Neuron density of the active site (AS) and the second binding site (BS2) of HIV-RT and ABL1, for all study combinations**

			**Decoys**	**Actives**	**EGF**
HIV-RT	AS (338.5Å ^3^)	ADvina	1.158	1.563	3.070
		Dock	0.718	1.731	2.065
	BS2 (957.4Å ^3^)	ADvina	1.692	1.349	0.976
		Dock	0.298	0.315	0.251
ABL1	AS (257.1Å ^3^)	ADvina	1.081	1.851	3.940
		Dock	1.412	3.003	6.410
	BS2 (615.5Å ^3^)	ADvina	2.587	2.244	2.600
		Dock	-	-	-

Inversely, in the BS2 with ADvina, the highest neuron density was observed with the decoys (1.692 neuron/Å ^3^), followed by the DUD-E active molecules (1.349 neuron/Å ^3^), then by the EGF fragments (0.976 neuron/Å ^3^). Dock outputs yielded low densities at the BS2 (Table 2).

#### ABL1

Cavities corresponding to ABL1’s AS (1’) and BS2 (6’) have volumes of 257.1Å ^3^ and 615.5Å ^3^, respectively. Neuron densities at the AS presented the same trend as that observed for HIV-RT. The EGF fragments had the highest densities (3.940 and 2.677 neuron/Å ^3^ with ADvina and Dock, respectively), followed by the DUD-E active molecules (1.851 and 1.254 neuron/Å ^3^ with ADvina and Dock, respectively), and finally the DUD-E decoys (1.081 and 0.590 neuron/Å ^3^ with ADvina and Dock, respectively; Table 2). For the BS2, the EGF fragments yielded the highest densities (2.600 neuron/Å ^3^ with ADvina). The DUD-E decoy showed the second highest neuron density at the BS2, followed closely by the active molecules, (2.587 and 2.244 neuron/Å ^3^, respectively; Table 2).

Overall, the EGF fragments yielded the highest neuron densities at the active sites regardless of the target and the docking software. Nevertheless, ADvina performed a better fitting of the identified BSs for both targets. Moreover, it is much faster than Dock. In the next step, we assessed our method performances on all targets in the DUD-E database using the EGF collection as probe library and ADvina as docking algorithm.

### Automatic BS identification

We automated the protocol identified with HIV-RT and ABL1: docking of the EGF collection with ADvina and called it “SOM-BSfinder”. We applied it on the 102 targets of the DUD-E. Regions of the SOMs presenting high U-values (see Methods section) were removed, and contiguous regions remaining on the SOM defined as the consensual clusters (CCs). The number of neurons per CC were used to sort them. The label 1 was attributed to the CC with the highest number of neurons, and so on.

**Figure 6:**
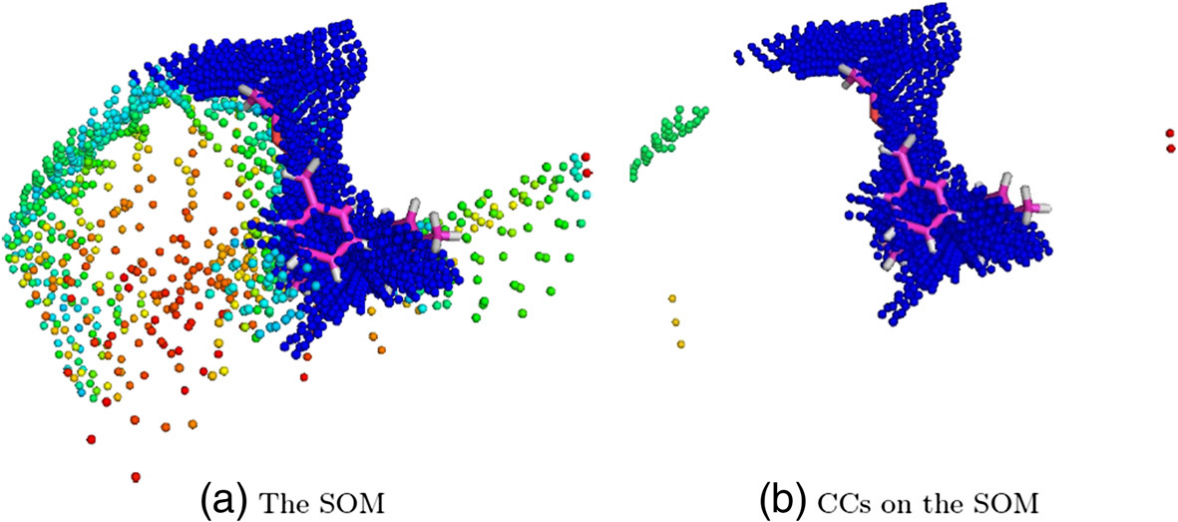
**Results obtained for the FKB1A target.** A test case where the AS is detected in the first CC with the maximal precision (fraction of overlapping atoms equal to 0.84). **(a)** The SOM obtained for FKB1A with the co-crystal ligand shown in pink licorice. **(b)** The CCs obtained are ranked with regards to their neuron densities and represented with a color gradient going from blue (most populated) to red (less populated). The first CC (dark blue) contains the 84% of the active ligand atoms (shown in pink licorice).

**Figure 7:**
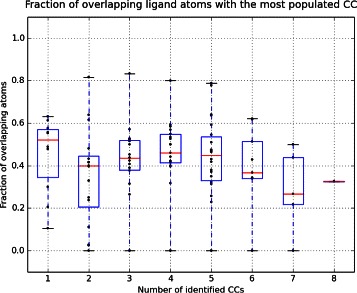
**Fraction of overlapping ligand atoms with the most populated consensual cluster (first CC).** Data is displayed according to the number of identified CCs.

The most populated CC, with label 1, was assumed to predict the AS while the co-crystallized ligand position was used to define the AS position. Hence, we calculated the fraction of ligand atoms contained in each SOM CC for each target. A ligand atom is considered “inside” a CC if it is located within a distance equal or superior to the radius of that CC (see Methods and Additional file 1: Figure S3). The average fraction of overlapping atoms was equal to 40% and 44% with the first CC and the first three CCs, respectively. The maximal fraction, equal to 84%, was observed at the first CC for the target FKB1A (Figure 6). If no precision criterion is applied on these fractions, SOM-BSfinder was able to detect atoms of the ligand within the most populated CC in 90% of the cases, and within one of the three most populated CCs (Top3) in 99% of the cases (101 targets, see Additional file 1: Table S3). SOM-BSfinder failed to detect the AS for only one target (XIAP). The distribution of these fractions is represented in Figure 7 according to the number of identified CCs.

**Figure 8:**
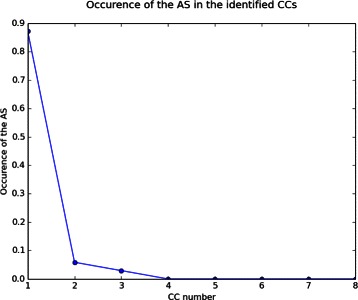
**Occurrence of the AS at the different CCs identified.** Cases where the AS is overlapping only one CC (98 cases, 96% of the targets) are considered for this plot. The first CC accounts for 87% of the cases, the second CC accounts for 6% and the third CC account for 3%. Zero occurrence for the AS was detected beyond the third CC.

To better evaluate the accuracy of SOM-BSfinder in detecting the AS, we calculated at which frequency the ligand atoms were found in SOM CC number *n* (Figure 8). To remain stringent, detection was considered as failed if the ligand overlapped two or more SOM CCs (3 targets: ACE, DRD3 and PLK1) or with no SOM CC (XIAP; hence, sum of frequency ≈ 96%). The AS was identified within the first most populated CC in 87% of the cases, and within the second or the third most populated CC in less than 9% of the cases. Beyond the third most populated CC, no overlapping was observed with the ligand atoms (Figure 8).

We calculated the success rate (SR) of SOM-BSfinder. For that, we consider that the AS was successfully identified if the fraction of ligand atoms within the radius *r*_*CC*_ (see Equation (2), Additional file 1: Figure S3) from SOM CC points was superior to a precision threshold of 0.25. SOM-BSfinder showed SR values of 90% when the Top3 CCs were considered, and 82% for the first CC alone (Table 3, first line). Then, we compared SOM-BSfinder performances to other energy-based/probe-mapping methods (FTSite [19], Q-SiteFinder [21,22] and SiteHound [27]), based on their success rates (SRs). For that, we had to adapt the precision and radius cutoffs to match those used by the authors of the concerned programs. FTSite [19] and Q-SiteFinder [21,22] consider that the AS was successfully identified if the fraction of ligand atoms within 1.6 Å of SOM CC points was superior to 0.25. In contrast, SiteHound uses a cutoff distance of 2.0 Å, a fraction superior to 0.15 and only consider heavy atoms. The results for the different methods and SOM-BSfinder with the respective parameters are shown in Table 3. In this specific context and with regards to the fact that different datasets were used by the described methods, SOM-BSfinder outperformed all three methods (see Additional file 1: Table S4).

**Table 3:** **Success rate values for SOM-BSfinder and other probe-mapping methods**

**Precision**	**Method**	**Atom type**	**Radius (Å)**	**Top3 SR**	**Top1 SR**
25%	SOM-BSfinder	all	[0.5-0.8]	90%	82%
25%	SOM-BSfinder	all	1.6	97%	88%
	FTSite ^(∗)^	all	1.6	97%	80%
	Q-SiteFinder	all	1.6	90%	71%
15%	SOM-BSfinder	heavy	2.0	98%	89%
	SiteHound	heavy	2.0	80-84%	–

In the first section, SOM-BSfinder performances with its defaults parameters. In the second section, default parameters of FTSite [21,22] and Q-SiteFinder [19] were used; (*) Values for FTSite were calculated on a set of 35 targets [22]. In the last section, default parameters of SiteHound [27] were used.

### Chemical descriptors

We tested if we could use information on the ligand chemical composition to provide information on relevant chemical groups, in addition to the fact that they refined the acuity of the active site identification.

We made this analysis on HIV-RT and ABL1, the benchmarks used to setup the method.

**Figure 9:**
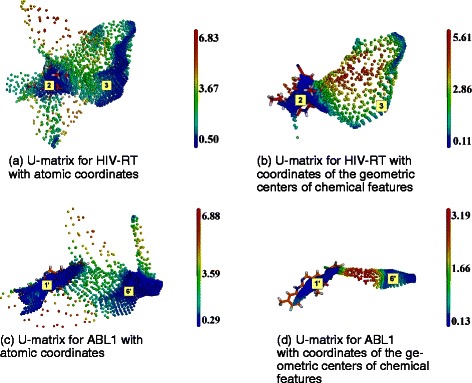
**SOM analysis of docking results obtained with ADvina with atomic coordinates as input vectors for HIV-RT (a) and ABL1 (c); and with the coordinates of the geometric centers of the chemical features as input vectors for HIV-RT (b) and ABL1 (d).** Labels (2), (3), (1’) and (6’) correspond to cavity numbers used in Figure 1. They designate the AS and BS2 of HIV-RT and ABL1, respectively.

We used the Morgan fingerprints as chemical descriptors (see Methods). They describe the molecule chemistry through an inventory of each atom environment, which can be viewed as local chemical groups or moieties. Their application gave higher neuron density and consensus for both HIV-RT and ABL1 (Figure 9), thus refining the BS geometrical definition.

To evaluate the insight provided with this approach, we calculated the enrichment of known “active features”. The latter term denotes chemical substructures observed in the active ligands provided by the DUD-E database. The enrichment in “active features” in the EGF collection *E*(*E* *G* *F* *d*), (Equation (3)), their presence in the AS, *E*(*A* *S*), (Equation (4)), and conversely their absence in the AS, $E(\overline {AS})$, (Equation (5)), are reported in Additional file 1: Table S5. For both targets, the proportion of active features docked in AS was larger than the proportion of active features that never docked in AS ($E(AS)/E(\overline {AS})$ is 4.64 and 4.44 for HIV-RT AS, and ABL1, respectively). Similarly, the proportion of active features docked in AS was also higher than the proportion of active features in the docked fragments (*E*(*A* *S*)/*E*(*E* *G* *F* *d*) is 2.85 and 2.87 for HIV-RT and ABL1, respectively, see Additional file 1: Table S5).

We calculated the sensitivity and the specificity of the method as described in Equations (6) and (7). For both targets, the sensitivity was moderate, whereas the specificity was high (0.49 and 0.85, respectively, for HIV-RT and 0.46 and 0.86, respectively, for ABL1 (Table 4)). The ratios *S* *e*/(1−*S* *p*) were higher than 1 in both cases (3.67 for HIV-RT and 3.28 for ABL1). We also calculated the Z-score of the sensitivity and the specificity values by comparison to randomized data (see Methods). The very high Z-score values obtained (Z-score ≥20, see Additional file 1: Table S6) show that these results are significantly away from a random distribution of the features over the identified CCs. Interestingly, the ratios *S* *e*/(1−*S* *p*) are close to 1 for both randomized tests, hence confirming lack of information content. *Se* seems to be more affected than *Sp* by the randomized test, suggesting that the sensitivity is, for those targets, the factor yielding a higher *S* *e*/(1−*S* *p*) ratio and, thus, the discriminating power of the method.

**Table 4:** **Sensitivity (Se) and specificity (Sp) values obtained for test targets HIV-RT and ABL1**

	**Se**	**Sp**	**Se/(1-Sp)**
HIV-RT	0.49	0.85	3.67
ABL1	0.46	0.86	3.28

## Discussion

In this work, we presented and evaluated a method for the identification of binding sites (BSs) based on docking and Self-Organizing Maps (SOMs). Binding site identification is essential in the process of structure-based drug discovery, but remains a highly complex task and an active area of research.

Our method bears similarities to probe-mapping approaches, but we took advantage of existing docking algorithms [44] to directly screen small molecule or fragment libraries. This allowed us to use entire molecules for the analysis. In contrast, classical probe mapping approaches use atoms or small chemical groups [17,18,20,68,69] to map a protein surface. The diversity of the probe library made it possible to take into account simultaneously shape, volume and the chemical composition of the protein surface in a more detailed way. Importantly, our method does not require any prior knowledge on active ligands, but actually identifies promising moieties.

To calibrate the method, we used 12 different “combinations” of protein targets (HIV-RT and ABL1), docking programs (ADvina and Dock) and ligand libraries (DUD-E active molecules, DUD-E decoys and EGF collection). The method readily identified the experimentally known ASs regardless of the target, the docking algorithm and the chemical library. For both targets, we could also identify a relevant second BS, a known allosteric site of HIV-RT [66], and an activation site controlling ABL1 catalytic activity [67,70]. The identified BSs appeared as dense and homogeneous regions of the SOMs.

The consistency of the results obtained with various different conditions suggests that the method is robust and applicable to different types of binding surfaces, ligands, and docking programs. Nevertheless, some combinations (docking algorithm, chemical library) appeared to perform better than others. The EGF fragments turned out to be the best probe library for our method, surpassing the dedicated DUD-E active molecules. This indicates that prior knowledge on the ligand is less important than the relevance of the probe library, possibly its chemical diversity and the moderate size of its components. ADvina gave a finer density and homogeneity compared to Dock. The energy grid calculation is a key step before the actual docking. ADvina uses AutoGrid [71] while Dock uses *grid* [72]. As far as we know, there is no study in binding site identification based on *grid* [72]. By contrast, AutoGrid is used by two successful probe-mapping/energy-based algorithms; AutoLigand [18] and SiteHound [27]. ADvina docking scores were the most favorable at the AS, and permitted to better discriminate it against BS2 for both targets. Conversely, Dock scores were not able to differentiate AS, BS2 and regions on the protein surface for HIV-RT. Finally, the U-values and the neuron density proved more reliable in identifying binding sites in general.

To assess the accuracy of our BS identification method, we automated it using EGF fragments as probe library and ADvina as docking algorithm. We called this automated approach SOM-BSfinder. The evaluation of the density and homogeneity of neurons on the 3D SOM allow to directly identify consensual clusters (CCs) ranked according to their densities. No limit to the number of BSs is required. The user may consider all identified CCs with respect to prior knowledge of the target, if available. Nevertheless, SOM-BSfinder was able to detect the AS exclusively among the Top3 CCs in 96% of the cases, and in the first CC in 87% of the cases (Figure 8). The average precision of the BS identification is 44% and 40% for the Top3 CCs and the first CC, respectively.

With a precision threshold of 25% to define success in identifying the AS, the success rate (SR) of SOM-BSfinder was equal to 90% and 82% for the Top3 CCs and first CC, respectively. It compared favorably with other probe-mapping/energy-based methods. We compared it with SiteHound [27] which also used AutoGrid for grid calculation for a carbon probe. This grid is used to identify three favorable BSs at the protein surface. These sites are then targeted for the docking of one ligand molecule with AutoDock 4 [28]. SiteHound achieved a success rate between 80 and 84% for the first three BSs. Using the same success criteria than SiteHound (radius = 2.0Å and precision = 15%), SOM-BSfinder achieved a success rate of 98%.

We also compared SOM-BSfinder to Q-SiteFinder [19] and FTSite [21,22]. Q-SiteFinder is very similar to SiteHound, but used the GRID [16] algorithm for grid calculation. On a set of 35 targets, with a radius fixed to 1.6Å and a precision threshold of 25%, it achieved 90% and 71% of success rates for the Top3 BSs and first BS, respectively. FTSite was tested on the same target set using the same parameter values [22]. It achieved 97% and 80% of success rates, respectively. We tested SOM-BSfinder with these values and obtained 97% and 88% of success for the Top3 CCs and Top1 CC, respectively. Thus, SOM-BSfinder is either as good or better than the three methods used for comparison. One should note that these results were obtained on different datasets, except for FTSite and Q-SiteFinder.

A major difference between SOM-BSfinder and the other methods is the probe library: SiteHound used a carbon atom probe, Q-SiteFinder used a methyl probe and FTSite used 16 organic probes with an average size of 4.3 heavy atoms. FTSite achieved the best SR among these three methods. In contrast, SOM-BSfinder used 1500 fragment molecules as probes, and achieved an SR equal or superior to FTSite. This may be a direct result of the diversity of the probe library used. Moreover, SOM-BSfinder takes into account the size and shape of the fragments during docking, which is less meaningful when the probe accounts for less than 8 heavy atoms (FTSite).

The ABL1 target was among the 9 cases out of 102 where the AS was identified at the second CC (labeled (6’) in Figure 4) by SOM-BSfinder. Notably, the first CC corresponded to the BS2 previously defined (labeled (1’) in Figure 4). Interestingly, when the SOMs are visualized with the docking score projections (Figure 4), it becomes more intuitive to select the AS, thus inverting the ranking of the CCs. This shows that the ranking criterion by decreasing densities, a common way of identifying the AS [13,73] that performs remarkably well, can still be further refined. For example, some energy-based methods [19,27], rank the BSs by the cumulative energy of their probes, and incorporate the quality of the docking and the size of the cluster.

Use of chemical feature positions as input for the SOMs improved the characterization as well as the discrimination of the AS and the BS2. For both test targets HIV-RT and ABL1, the predicted AS fits the experimentally known one and is depicted by low U-values that reflect the homogeneity of the docking poses. The BS2 is characterized by a less distinct area in the SOM than the AS, with less favorable docking scores. Moreover, a larger discrimination of “active features” is found in the ASs, and the specificity is over 84%. This characteristic of the method could prove useful in predicting relevant substructures, and favoring hit discovery and optimization. It also readily provides a set of potentially active fragments for test in a drug design project.

## Conclusions

The present work presents a new method for binding site identification called SOM-BSfinder. It is a probe-mapping method that uses docking of a compound library to map the protein target surface. Atomic coordinates of the docked molecules are clustered using a Self-Organizing Map algorithm to generate a 3D map that reflects preferential binding positions on the protein surface. These positions constitute consensual clusters that define the favored binding sites of the probes. The method was calibrated on two test targets to identify the best conditions for optimal performances. In a second phase, a benchmark was performed on 102 proteins using AutoDock vina for docking the Enamine Golden Fragments collection. SOM-BSfinder achieved 90% of successful detection when the first three consensual clusters are retained, and 82% when only the first cluster is considered. Compared to existing method, our method achieved either equal or superior success. The last part of this work consists in the use of chemical decomposition, using the circular Morgan fingerprints, of the probes molecules instead of an atomic decomposition. This lead to a better fit and descrimination of the active sites. Moreover, these results could also be used to predict chemical moieties relevant to bioactivity.

A further advantage of our method is its high flexibility. In our hands, the combination of AutoDock Vina and the Enamine Golden Fragments collection gave the best predictions. Nonetheless, similar pipelines could be implemented with other docking programs, fragment libraries and/or clustering algorithm to better exploit the user’s knowledge and expertise on the targeted protein. Similarly, features other than the Morgan fingerprints can be employed to describe the ligand chemistry.

## Additional file

Additional file 1
**Additional file (SupplementaryInformations.pdf) contains figures and tables with their respective captions and descriptions.**

## Abbreviations

3D: Three dimensional
AA: Amino acid
ADvina: AutoDock vina
AS: Active site
BS: Binding site
BS2: Secondary binding site
CC: Consensual cluster
DUD-E: Database of useful decoys enhanced
EGF: Enamine golden fragments
Hbond: Hydrogen bonds
PCA: Principal component analysis
PDB: Protein data bank
Se: Sensitivity
SOM: Self-organizing map
Sp: Specificity
SR: Success rate
