# Supplementary material for: Identification of binding sites and favorable ligand binding moieties by virtual screening and self-organizing map analysis
Source: BMC Bioinformatics. 2015 Mar 21;16:93. doi: 10.1186/s12859-015-0518-z (PMC4381396; doi:10.1186/s12859-015-0518-z)
Supplement: Additional file 1 — Additional file (SupplementaryInformations.pdf) contains figures and tables with their respective captions and descriptions. [file 12859_2015_518_MOESM1_ESM.pdf]

Identification of binding sites and favorable ligand binding moieties by virtual screening and Self-Organizing Map analysis

Emna Harigua-Souiai, Isidro Cortes-Ciriano, Nathan Desdouits, Thérèse E. Malliavin,
Ikram Guizani, Michael Nilges, Arnaud Blondel, Guillaume Bouvier*

February 5, 2015

Additional Tables

Target	PDB entry	Cav number	Cav ids
pa2ga	1kvo	8	1 2 3 5 14 15 16 19 30
hmdh	3ccw	6	3 6 9 12 13 15
braf	3d4q	5	2 3 4 5 6
reni	3g6z	4	1 3 7 8
prgr	3kba	4	1 2 3 4
pgh2	3ln1	4	6 7 11 13
glcm	2v3f	4	2 6 7 11
esr2	2fsz	4	3 8 9 11
dpp4	2i78	4	15 17 20 24
cxcr4	3odu	4	3 4 6 9
cp3a4	3nxu	4	1 2 3 5
mk01	2ojg	3	2 4 5
kpcb	2i0e	3	1 10 11
kith	2b8t	3	1 8 9
hivrt	3lan	3	2 3 6
esr1	1sj0	3	1 4 5
drd3	3pbl	3	1 4 5
cp2c9	1r9o	3	3 5 6
aofb	1s3b	3	2 5 11
adrb1	2vt4	3	1 6 9
aces	1e66	3	2 3 7
vgfr2	2p2i	2	1 2
thrb	1ype	2	2 3
thb	1q4x	2	1 2
tgfr1	3hmm	2	1 2
src	3el8	2	5 6
sahh	1li4	2	6 8
pyrd	1d3g	2	10 11
pygm	1c8k	2	6 8
pparg	2gtk	2	3 5
ppard	2znp	2	1 10
ppara	2p54	2	1 2
pgh1	2oyu	2	2 3
parp1	3l3m	2	3 4
nram	1b9v	2	1 3
mcr	2aa2	2	2 3
lck	2of2	2	3 6
jak2	3lpb	2	4 5
inha	2h7l	2	1 6
gria2	3kgc	2	1 2
gcr	3bqd	2	1 3
fgfr1	3c4f	2	3 4
dhi1	3frj	2	2 5
bace1	3l5d	2	1 2
andr	2am9	2	5 6
ampc	1l2s	2	3 5
adrb2	3ny8	2	1 2
ace	3bkl	2	1 3
abl1	2hzi	2	1 6

Table S1 DUD-E targets sub-domains containing at least two cavities (detected with mkgrid) with a volume superior to 100 Å^3. The last column contains the labels obtained with mkgrid for the detected cavities.

HIV-RT	Cavity label	1	**2**	**3**	4	5	6
	Cavity volume (Å^3)	15.4	**338.5**	**957.4**	83.8	34.8	294.9
	Neuron density (ADvina)	0.260	**3.070**	**0.976**	0.0	0.0	0.058
	Neuron density (Dock)	0.0	**2.065**	**0.251**	0.465	0.0	0.0
	Cavity label	7	8	9			
	Cavity volume (Å^3)	15.4	15.4	15.4			
	Neuron density (ADvina)	1.494	1.494	0.0			
	Neuron density (Dock)	0.0	0.0	0.0			
ABL1	Cavity label	**1'**	2'	3'	4'	5'	**6'**
	Cavity volume (Å^3)	**257.1**	15.4	52.8	46.8	15.1	**615.5**
	Neuron density (ADvina)	**3.940**	0.0	0.0	0.043	0.861	**2.600**
	Neuron density (Dock)	**6.410**	0.0	0.0	0.0	0.0	**0.0**
	Cavity label	7'	8'	9'	10'	11'	12'
	Cavity volume (Å^3)	79.6	15.1	22.1	46.6	89.5	37.6
	Neuron density (ADvina)	0.0	0.0	0.0	0.0	0.0	0.0
	Neuron density (Dock)	0.0	0.0	0.0	0.0	0.0	0.0

Table S2 Cavities detected by mkgrid on HIV-RT and ABL1. Their label, volume and neuron density (neuron/Å^3) were calculated with the SOMs obtained with the EGF library. Cavities corresponding to AS and BS2 are in bold for each target.

Method	Set size	Success rate (SR)	
		Top3	Top1
SOM-BSfinder	102	99%	90%
FTsite	48	98%	94%
	35	97%	97%
Q-siteFinder	134	86%	80%
SiteHound	77	95%	77%
AutoLigand	187	–	73%

Table S3 Success rates for SOM-BSfinder and other energy-based algorithms when precision threshold is set to zero. These results are consistant with those obtained with various precision thresholds. SOM-BSfinder outperformed Q-siteFinder, SiteHound and AutoLigand, but presented lower success rates compared to FTSite.

Target	SOM-BSfinder	SiteHound	FTSite
HIV-RT	1	3	1
ABL1	2	2	3
RENI	1	2	1,2,3
BRAF	1	2,3	1
HMDH	1	6	1,2
PA2GA	1	1,2	1,2

Table S4 Active Site rank calculated for 6 targets in the DUD-E, chosen in different categories (table 1), using SOM-BSfinder, SiteHound and FTSite. With ABL1, SOM-BSfinder and SiteHound ranked the AS as the second position and FTSite ranked it in the third one. Otherwise, SOM-BSfinder ranked the AS as the first CC, which is not the case for FTSite and SiteHound. The latter algorithms showed more variability in the ranking.

	E(EGFd)	E(AS)	E($\overline{AS}$)	E(AS)/E(EGFd)	E(AS)/E($\overline{AS}$)
HIV-RT	0.08	0.22	0.05	2.85	4.64
ABL1	0.06	0.18	0.04	2.87	4.44

Table S5 The enrichments in "active features" of the docked fragments (E(EGFd)), the AS (E(AS)) and the complementary of AS (E($\overline{AS}$)) for the test targets HIV-RT and ABL1.

Target	Metric	Value	μ	σ	Z-score
HIV-RT	Se	0.49	0.17	10^{-2}	23
	Sp	0.85	0.82	10^{-3}	23
ABL1	Se	0.46	0.16	10^{-2}	20
	Sp	0.86	0.84	10^{-3}	20

Table S6 Z-scores of the sensitivity (Se) and specificity (Sp) values obtained with the chemical features decomposition analysis for the test targets: HIV-RT and ABL1. For the sensitivity, we simulated a random sampling over features docking in the AS (F_{AS}) 1 million times. In a perfect scenario, all the active features (F_A) would dock in the AS, giving a sensitivity equal to 1. In the worst scenario, none of the active features would dock in the AS. The resulting samples were normally distributed $N(\mu, \sigma)$. The Z-score is the distance in terms of σ between the "experimental" value and the mean μ of the normal distribution. We did the same for the sensitivity, sampling randomly over features that would never dock in the AS ($\overline{F_{AS}}$).

Additional Figures

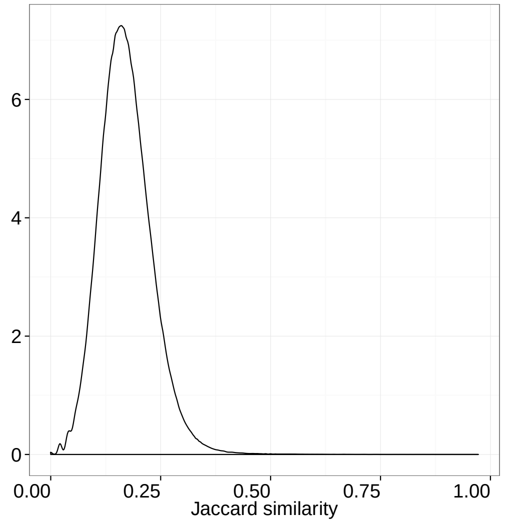

Figure S1 Distribution of the Jaccad distance over the EGF fragments. The jaccard pairwise distance between binary fingerprints of the EGF compounds was computed. The lower the jaccard distance between two compound fingerprints, the lower the similarity between them. The distribution indicates low jaccard pairwise distances between the elements of the EGF collection. Based on this analysis, we concluded that the EGF collection presents a high chemical diversity in spite of its small size (1500 fragments).

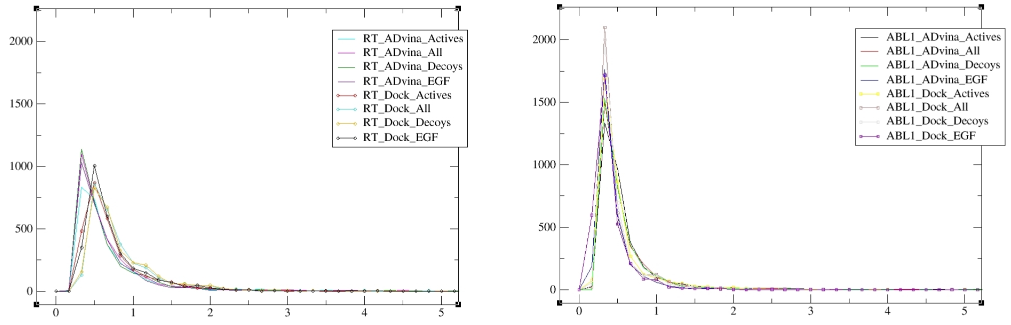

Figure S2 Distributions of the distances of the input vectors to their representative neurons on the SOMs.

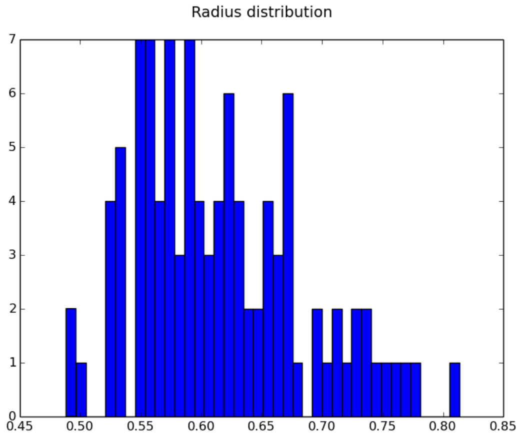

Figure S3 Distribution of the radius. mean=0.6, min=0.5, max=0.8.
